# Food Insecurity, Depression, and Race: Correlations Observed Among College Students at a University in the Southeastern United States

**DOI:** 10.3390/ijerph17218268

**Published:** 2020-11-09

**Authors:** Nicole Reeder, Pradtana Tapanee, Anna Persell, Terezie Tolar-Peterson

**Affiliations:** Department of Food Science, Nutrition, and Health Promotion, Mississippi State University, Starkville, MS 39762, USA; nr657@msstate.edu (N.R.); pt419@msstate.edu (P.T.); amp1074@msstate.edu (A.P.)

**Keywords:** food security, food insecurity, college students, depression, mental health

## Abstract

Food insecurity is common among college students in the United States and is associated with poorer health-related outcomes and academic performance. The aims of this study were to assess the prevalence of food insecurity at a large, public university in Mississippi, a state with the second highest rate of food insecurity in the nation, and to examine the associations between food insecurity, depression, and race in this group of students. Food security was measured using the United States Department of Agriculture Household Food Security Survey Module: Six-Item Short Form, and depression was measured using the Patient Health Questionnaire-9. In total, 131 students ages 18–24 participated in the study. Food insecurity was present in 38.2% of students surveyed. The odds of food insecurity were higher among African American students compared to Caucasian students (OR = 3.50, 95% CI: 1.38, 8.90). Students with very low food security had 4.52-times greater odds of having depression than food-secure students (*p* = 0.011, 95% CI: 1.42, 14.36). Neither body mass index nor body fat percentage were associated with food security status. Further research is needed on strategies to address the risk of depression among food-insecure college students and the racial disparity in food insecurity rates present among college students.

## 1. Introduction

Food insecurity is an economic and social condition marked by having an inadequate amount of food to live an active, healthy life [1]. In 2018, 11.1% of households in the United States were food insecure for at least part of the year [2]. In the state of Mississippi, the rate of food insecurity is above the national average at 15.9%, placing Mississippi second in the nation for its rate of food insecurity, only behind New Mexico which has a food insecurity rate of 16.8% [3]. Populations traditionally at a higher risk for food insecurity include single-parent households, households with children, households with single individuals living alone, and African American and Hispanic households [3]. More recently, there has been an increasing awareness of the presence of food insecurity in college campuses in the United States. The rate of food insecurity among college students is estimated to be approximately 43%, which is significantly higher than the national average of 11.1% of households [4,5]. College students who may be at a higher risk of food insecurity include those at two-year institutions, those of a racial or ethnic minority, LGBTQ students, students with prior military service, former foster youth, students with prior criminal convictions, students from low-income families, students receiving multiple forms of financial aid, and students who are financially independent from their parents [6,7].

Food security is an essential component to optimal health, and adults with food insecurity face a higher risk of nutritional deficiencies, obesity, hypertension, hyperlipidemia, and diabetes [8,9,10]. Food-insecure adults are additionally more likely to have a lower attained education level, not have health insurance, and be smokers, which all contribute to poorer health outcomes [11]. Individuals with food insecurity also have greater annual healthcare expenditures, which adds up to an estimated extra USD 77.5 billion in additional healthcare costs each year [12]. The health disparities experienced by food-insecure individuals are also intertwined with racial disparities in health outcomes and rates of food insecurity. In the United States, being of a minority race is associated with an increased risk for experiencing food insecurity and an increased risk for development of chronic diseases. African American and Hispanic headed households have greater than average rates of food insecurity [3] and are also at a greater risk for type 2 diabetes [13], hypertension [14], and cardiovascular disease [15]. Looking at Mississippi, the state’s African American population has the highest mortality rate due to heart disease, hypertension, stroke, and diabetes [16], in addition to having a significantly greater prevalence of food insecurity compared to Mississippi’s Caucasian population [17]. These data consistently demonstrate that food insecurity, race, and health outcomes are all closely related.

In addition to physical health, food insecurity is also associated with mental health. The relationship between food insecurity and poor mental and emotional health is believed to be bidirectional, where food insecurity increases the risk of poor mental health, and poor mental health also increases the risk of food insecurity [18]. Food insecurity has been associated with depression and mental distress, especially in individuals already under higher stress and those socially isolated [19,20]. In young adults, food insecurity may also be associated with suicidal ideation, substance use problems, anxiety or panic disorder diagnoses, and poor sleep outcomes [21,22]. This is concerning for the college student population where both stress and food insecurity are known to be highly prevalent. College students are in a unique stage of life, and food-insecure college students may face additional challenges that can exacerbate mental distress such as worrying about grade point average (GPA) and balancing school with work and extracurricular activities. In a study of university students from New Jersey, food insecurity increased the odds of a student being in the lower 10% for GPA and decreased the odds of a student being in the upper 10% for GPA [23]. In college students from California, food insecurity has been associated with poor health behaviors such as limited physical activity, consuming fewer servings of fruits and vegetables, and not getting enough sleep [24]. In another study that looked at college students from Florida, Maine, Tennessee, Alabama, South Dakota, Kansas, New York, and West Virginia, food-insecure students had poorer sleep, higher stress, and a higher odds of disordered eating behaviors compared to food-secure students [25]. Clearly, food-insecure college students face challenges that extend far beyond mealtime.

Current evidence suggests food insecurity is prevalent on college campuses across America and that food insecurity is linked to poor health outcomes and academic difficulty. Despite Mississippi having one of the highest rates of food insecurity in the nation and having many racial health disparities, there is currently limited knowledge on the state of food insecurity on college campuses in Mississippi. Additionally, there remains limited or inconsistent knowledge on how mental distress manifests in food-insecure college students. Thus, the primary objectives of this study were to examine the relationships between food security, depression, and race among college students at a large public university in Mississippi.

## 2. Materials and Methods

### 2.1. Study Design

This was a cross-sectional study of college students, ages 18–24, enrolled at Mississippi State University. Data were collected between October 2019 and April 2020. Subjects were recruited via email, flyers, and in-classroom announcements. Inclusion criteria included being age 18 or older, being able to read and respond in English, and willingness to complete all parts of the study. Interested subjects were required to make one visit to a campus laboratory where they completed body composition testing and a series of computer-based surveys in Qualtrics that asked questions related to demographics, food security, mental health, and alcohol use. The study was conducted in accordance with the Declaration of Helsinki, and the protocol was approved by the Ethics Committee of Mississippi State University (IRB 17-025). All subjects provided their written informed consent prior to beginning any part of the study.

### 2.2. Measures

#### 2.2.1. Food Security

Food security status was measured using the United States Department of Agriculture’s U.S. Household Food Security Survey Module: Six-Item Short Form [26]. This survey uses a subset of questions from the standard 18-item Food Security Survey Module. The Six-Item Short Form is a reliable substitute for the 18-item Food Security Survey Module and has a lower respondent burden. Limitations of the Six-Item Short Form are that it does not ask about conditions of children in the household, nor does it measure the most severe levels of food insecurity [27]. However, for our study population of college students, these limitations were deemed to have minimal impact on the measurement of food security.

The food security survey was self-administered on the Qualtrics platform. Questions five and six of the survey were combined into one question in accordance with United States Department of Agriculture (USDA) guidance. Affirmative responses were summed to obtain a total score for level of food security. Subjects with a raw score of 0–1 were classified as having high or marginal food security, scores of 2–4 were classified as low food security, and scores of 5–6 were classified as very low food security. For some analyses, food security was made a dichotomized variable by grouping subjects with a total score of 0–1 as food secure and subjects with a total score of 2–6 as food insecure, per USDA definitions.

#### 2.2.2. Depression

Depression was assessed by administering the Patient Health Questionnaire-9 (PHQ-9) to subjects. The PHQ-9 is a self-report depression screening tool that is based on the nine items reflective of major depressive disorder in the Diagnostic and Statistical Manual of Mental Disorders, Fourth Edition (DSM-IV). It is a reliable and valid measure of depression severity [28]. Each question is scored from “0” (not at all) to “3” (nearly every day) with the final questionnaire scores ranging from 0–27. Final scores were categorized by depression severity based on questionnaire guidelines. Scores of 1–4 were classified as minimal depression, 5–9 as mild depression, 10–14 as moderate depression, 15–19 as moderately severe depression, and 20–27 as severe depression.

#### 2.2.3. Alcohol Use and Body Composition

Data on alcohol use and body composition were also collected to further examine health and wellness among food-secure and food-insecure college students. All subjects completed the Alcohol Use Disorders Identification Test (AUDIT) which is a tool to screen for excessive drinking and alcohol use disorders. The AUDIT survey was self-administered on the Qualtrics platform. Trained research assistants then measured each subject’s height with a touchless digital stadiometer (Detecto SONARIS) and measured each subject’s body mass index (BMI) and body fat percentage with a bioelectrical impedance analysis scale (Tanita).

### 2.3. Statistical Analysis

All statistical analyses were performed in SPSS, version 26 (IBM, Armonk, NY, USA). Subjects with missing data for any of the variables of interest were excluded from the final analysis. Descriptive statistics were calculated for all variables and are presented as means and standard deviations or counts and percentages. Food security status was dichotomized into food secure and food insecure based on the total score obtained in the food security questionnaire, with scores of 0–1 defined as food secure, and scores of 2–6 defined as food insecure. Characteristics of students by food security category were compared using independent sample t-tests for continuous variables and Chi square tests for categorical variables. For the categorical variables of BMI (Table 1) and depression scoring (Table 2), Fisher’s exact tests were used, due to the frequency per cell being < 5. Binary logistic regression was carried out and odds ratios calculated for the associations between race and food security, and depression and food security. To further examine the relationship between food security and degree of depression, a one-way ANOVA and Tukey post-hoc test was run using all three categories of food security: high food security, low food security, and very low food security. All tests were two-tailed and *p*-values less than 0.05 were considered statistically significant.

## 3. Results

### 3.1. Participant Characteristics

After removing subjects with missing data, the final data analysis included 131 college students. The majority of subjects were female (*n* = 95, 72.5%) and Caucasian (*n* = 93, 71.0%), with an average age of 19.77 ± 1.68 years (Table 1). Compared to demographic data for Mississippi State University students, female subjects were overrepresented in our study sample, but racial demographics were comparable to the university’s student enrollment. Underclassmen made up more than half of the student subjects with 62.6% of students in this study being either freshmen or sophomores. Most students (62.3%) also reported having a meal plan. By BMI, 59.5% of students were of a normal or healthy weight, and 33.6% of students were overweight or obese.

### 3.2. Food Security

Food insecurity was present in 38.2% of all students surveyed. Of the food-insecure students, 64.0% were classified as having low food security, and 36.0% were classified as having very low food security. Food insecurity was not associated with age or sex but was significantly correlated with race (χ^2^ = 7.43, *p* = 0.024). In African American students, 62.5% of students reported food insecurity, whereas in Caucasian students, 32.26% reported food insecurity. The odds of being food insecure were 3.5-times greater for African American students compared to Caucasian students (95% CI: 1.38, 8.90). There were no significant differences in BMI, body fat percentage, or alcohol use between food-secure and food-insecure students, nor were year in school or having a meal plan associated with food security status.

### 3.3. Depression

The mean PHQ-9 score for all participants was 5.85 ± 4.93, with scores ranging from 0 to 22. Among all students surveyed, 19.1% had PHQ-9 scores in the moderate to severe depression range. Food-insecure students scored significantly higher than food-secure students on the PHQ-9 depression assessment (*p* = 0.001, Table 1) and food security status was additionally associated with PHQ-9 depression categories (*p* = 0.004, Table 2). Moderate to severe depression was present at a significantly higher rate in food-insecure students compared to food-secure students, with 12.3% of food-secure students reporting moderate to severe depression, and 30.0% of food-insecure students reporting moderate to severe depression (χ^2^ = 6.24, *p* = 0.012). Having moderate to severe depression was also associated with higher odds of being food insecure compared to having minimal to mild depression (OR: 3.04, 95% CI: 1.24, 7.46).

**Figure 1:**
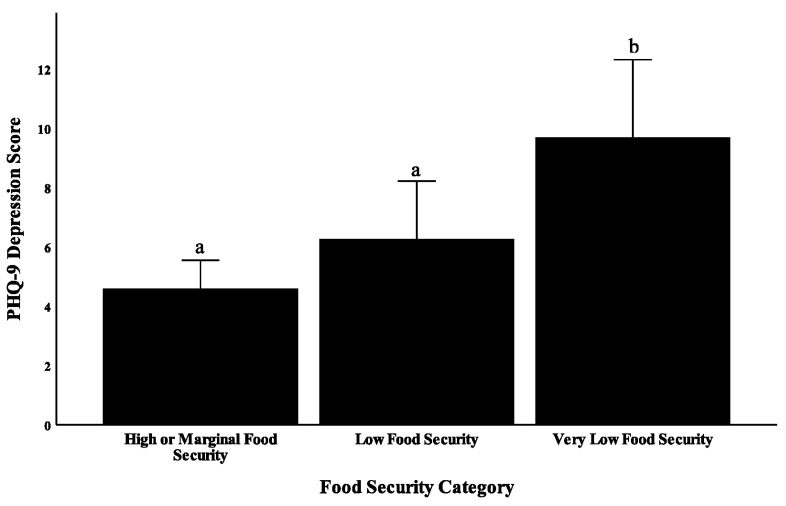
Food security and depression in college students at Mississippi State University. Students with very low food security scored significantly higher on the PHQ-9 than students with low food security (*p* = 0.016) and students with high or marginal food security (*p* < 0.001). Different letters on the graph indicate a statistically significant difference with a *p*-value less than 0.05 (ANOVA with Tukey post-hoc test).

Food-insecure students were divided into students with low food security, and students with very low food security to further examine the association between food insecurity and severity of depression. A significant relationship between degree of food security (high/marginal food security, low food insecurity, and very low food insecurity) and depression severity was observed (F = 10.77, *p* < 0.001, Figure 1). Students with high or marginal food security had an average PHQ-9 score of 4.7, students with low food security had a mean score of 6.4, and students with very low food security had a mean score of 10.2, suggesting that severity of food insecurity and severity of depression may be correlated in college students. Furthermore, results from binary logistic regression showed that students with very low food security have 4.52-times greater odds of having depression than food-secure students (*p* = 0.011, 95% CI: 1.42, 14.36). Students with low food security did not have a significantly greater odds of having depression compared to food-secure students (*p* = 0.104, OR: 2.37, 95% CI: 0.84, 6.69).

Certain symptoms of depression were significantly more prevalent in the food-insecure group, including feeling down, hopeless, or depressed, trouble sleeping, feeling tired or having little energy, poor appetite or overeating, and feeling bad about oneself. When comparing scores per question, both food-secure and food-insecure students scored highest for “feeling tired or having little energy”, with the next highest scored questions by both groups being “trouble falling or staying asleep, or sleeping too much”, and “poor appetite or overeating.” There was no significant difference between food-secure and food-insecure students in self-reported difficulty at work, home, or in getting along with other people as a result of these problems.

## 4. Discussion

In the present study, we sought to examine not only the prevalence of food insecurity at a university in Mississippi—one of the highest-ranking states for food insecurity in the United States—but also how food insecurity correlates with mental health. The prevalence of food insecurity found in this cohort of students from Mississippi State University was 38.2%. In comparison, a recent study on food security conducted across eight other land grant institutions in the United States found a food insecurity rate of 19% [25]. The results of this study thus suggest that just as Mississippi has a higher rate of food insecurity than most other states, Mississippi’s college students may also face a higher rate of food insecurity than other college students across America. Studies on food insecurity rates at other post-secondary schools in Mississippi are limited; however, a 2017 study from the University of Mississippi found 46.1% of their students to be food insecure, [29] which suggests that this is a widespread problem throughout the state. Additionally, 47.86% of 2017 high school graduates in Mississippi who went on to enroll in an in-state community college within 16 months of graduation were classified as economically disadvantaged, and 14.72% of graduates who went on to enroll in an in-state four-year public university were economically disadvantaged [30]. Lastly, data from the 2015 Mississippi Behavioral Risk Factor Surveillance System found that two out of every five Mississippi adults are food insecure, which further indicates the likelihood of food insecurity rates in college students being higher than average state-wide [17].

There was a significant racial disparity in food insecurity rates present in our subject population which is also consistent with previous studies of food insecurity in universities across the United States [25,31]. Here, the odds of being food insecure were 3.5-times greater for African American students compared to Caucasian students. This trend can be observed in both college student populations and in household data for food insecurity across the United States. In the state of Mississippi, the prevalence of food insecurity for African American adults is much higher than it is for Caucasian adults, with African Americans having a food insecurity rate of 53.7%, and Caucasian adults having a food insecurity rate of 36.4% [17]. Looking at data for the United States as a whole, from 2001 to 2016, food insecurity rates for both non-Hispanic black and Hispanic households were at least twice those of non-Hispanic white households, the reasons for which are complex and intertwined with gaps in rates of poverty, unemployment, incarceration, and disability [32]. Ultimately, addressing the racial inequity in food insecurity rates in college students requires addressing the issue on a broader level that considers the role of structural racism in the United States and gaps in the safety net provided by federal food and nutrition assistance programs.

Regarding depression in this group of college students, nearly 20% of students surveyed had PHQ-9 scores suggestive of moderate to severe depression, highlighting the importance of access to mental health services on college campuses. A number of recent studies have indicated that depressive symptoms co-exist with food insecurity in college students [7,33,34], and here, we confirm and extend these findings. Our results suggest that the severity of food insecurity is associated with the severity of symptoms of depression. Students who were the most food insecure (those with very low food security) had a mean PHQ-9 score of 10.2, which is suggestive of a moderate level of depression. On the other hand, food-secure students had a mean PHQ-9 score of 4.7, which suggests minimal or very mild depression. Whether food insecurity leads to depression or depression leads to food insecurity in college students remains in question. It is likely that this relationship is bi-directional, but because most studies that have been conducted on this topic have been cross-sectional in nature, this remains to be confirmed. In this study, we also found that college students who were food insecure had depression that was marked especially by symptoms of: (1) feeling down, hopeless, or depressed, (2) trouble sleeping, (3) feeling tired or having little energy, (4) poor appetite or overeating, and (5) feeling bad about oneself. Food insecurity in adults is associated with overweight and obesity, an increased risk for chronic health conditions, and insufficient dietary intake [35,36,37]. When coupled with depression, these nutrition-related concerns may be exacerbated further by the common depressive symptoms that were found in this college student population such as poor appetite, overeating, feeling tired or having little energy, and trouble sleeping. These same symptoms of depression may also contribute to mediating the relationship between food insecurity and poor academic performance [34]. These results are also in line with findings from a 2020 study of graduate students in the northeastern United States which found that food-insecure graduate students faced significantly greater rates of depression, stress, and anxiety [38]. Ultimately, these results highlight the imperative need to recognize food insecurity as a key social determinant of health for college students.

Recognizing food insecurity can be challenging, and a lack of consistency in measuring methods across studies can make comparisons difficult. A strength of this study is the use of the USDA six-item screener which has a low subject burden, and because it is a validated and commonly used measure, results across studies can be more easily compared. A limitation of this study is that it did not measure student GPA, and there is some evidence that psychosocial health may be a mediator of the relationship between food insecurity and lower GPA in college students [34]. We additionally did not collect data on students’ living situations or whether or not they receive any financial aid, both of which have previously been linked to food security risk [39]. The cross-sectional nature of this study also limits what conclusions can be drawn. Future studies may consider longitudinal or experimental study designs to help further explain the relationships between food security, mental health, and academic performance in college students. Lastly, the relatively small sample size of this study may not have been adequate to capture all potential differences between food-secure and food-insecure students. For example, food insecurity was not associated with BMI or body fat percentage in this study, though many previous studies have found food insecurity to be associated with obesity [24,37,40,41,42].

## 5. Conclusions

Collectively, the results of this study and the larger body of literature suggest that college students who are food insecure are facing a wide array of interrelated challenges. In this study, nearly 40% of college students at Mississippi State University reported experiencing some degree of food insecurity. Food insecurity is common and must be addressed. Particular attention should be paid to minority students who may be at the greatest risk of food insecurity. Here, African American students had 3.5-times greater odds of being food insecure compared to Caucasian students. Students who are food insecure also had higher odds of experiencing depression. In response, campus food pantries, which are becoming increasingly common on university campuses, may wish to consider more targeted marketing of their services to the highest risk groups of students on campus. Campus food pantries may also be an important space for promoting mental health and counseling services that are available to students. Ultimately, further efforts in the form of health promotion programming and interventions are needed on college campuses to support food-insecure students.

## Figures and Tables

**Table 1:** Characteristics of the Study Group and Correlations with Food Security Status.

Variable	All Subjects (*n* = 131)	Food Secure (*n* = 81)	Food Insecure (*n* = 50)	*p*-Value ^1^
Age	19.77 ± 1.68	19.68 ± 1.86	19.92 ± 1.34	0.426
Sex, *n* (%)				0.765
Male	36 (27.5%)	23 (28.4%)	13 (26.0%)	
Female	95 (72.5%)	58 (71.6%)	37 (74.0%)	
Race/ethnicity, *n* (%)				0.024
Caucasian	93 (71.0%)	63 (77.8%)	30 (60.0%)	
African American	24 (18.3%)	9 (11.1%)	15 (30.0%)	
Other	14 (10.7%)	9 (11.1%)	5 (10.0%)	
Year, *n* (%)				0.534
Freshman	44 (33.6%)	31 (38.3%)	13 (26.0%)	
Sophomore	38 (29.0%)	24 (29.6%)	14 (28.0%)	
Junior	29 (22.1%)	16 (19.8%)	13 (26.0%)	
Senior	18 (13.7%)	19 (23.5%)	9 (18.0%)	
Graduate Student	2 (1.5%)	1 (1.2%)	1 (2.0%)	
Meal plan ^2^, *n* (%)				0.393
Yes	82 (62.6%)	53 (65.4%)	29 (58.0%)	
No	49 (37.4%)	28 (34.6%)	21 (42.0%)	
BMI, *n* (%)				0.088
Underweight	9 (6.9%)	8 (9.9%)	1 (2.0%)	
Normal weight	78 (59.5%)	43 (53.1%)	35 (70.0%)	
Overweight	31 (23.7%)	23 (28.4%)	8 (16.0%)	
Obese	13 (9.9%)	7 (8.6%)	6 (12.0%)	
Body Fat Percentage				
Males	19.10 ± 9.11	19.61 ± 9.21	18.20 ± 9.23	0.661
Females	27.14 ± 6.90	27.26 ± 6.82	26.95 ± 7.13	0.831
AUDIT score	4.04 ± 4.02	3.75 ± 3.18	4.50 ± 5.11	0.304
PHQ-9 score	5.85 ± 4.93	4.68 ± 4.09	7.74 ± 5.91	0.001

^1^ Statistical significance determined by independent samples t-tests (age, body fat percentage, AUDIT score, and PHQ-9 score), chi-square tests (sex, race, year, and meal plan), or Fisher’s exact tests (BMI). ^2^ As part of the demographics survey, students were asked whether they had purchased a student meal plan for the current school year. This question was presented as a binary variable with students holding any level of meal plan being a “yes” and students holding no meal plan being a “no.”.

**Table 2:** Patient Health Questionnaire-9 Results.

Variable	Food Secure *n* = 81	Food Insecure *n* = 50	*p*-Value ^1^
Depression Scoring, *n* (%)			0.004
Minimal depression (0–4)	48 (59.3%)	15 (30.0%)	
Mild depression (5–9)	23 (28.4%)	20 (40.0%)	
Moderate depression (10–14)	8 (9.9%)	8 (16.0%)	
Moderately severe depression (15–19)	1 (1.2%)	5 (10.0%)	
Severe Depression (20–27)	1 (1.2%)	2 (4.0%)	
Scoring per question, mean ± SD			
Little interest or pleasure in doing things	1.48 ± 0.67	1.70 ± 0.81	0.098
Feeling down, depressed, or hopeless	1.36 ± 0.58	1.86 ± 0.99	0.002
Trouble falling or staying asleep, or sleeping too much	1.89 ± 0.91	2.34 ± 1.12	0.018
Feeling tired or having little energy	2.21 ± 0.90	2.54 ± 0.93	0.047
Poor appetite or overeating	1.77 ± 0.94	2.22 ± 1.18	0.024
Feeling bad about yourself—or that you are a failure or have let yourself or your family down	1.36 ± 0.62	1.86 ± 1.03	0.003
Trouble concentrating on things, such as reading the newspaper or watching television	1.40 ± 0.68	1.64 ± 0.88	0.095
Moving or speaking so slowly that other people could have noticed. Or the opposite—being so fidgety or restless that you have been moving around a lot more than usual	1.19 ± 0.53	1.42 ± 0.79	0.065
Thoughts that you would be better off dead, or of hurting yourself	1.04 ± 0.25	1.16 ± 0.47	0.091
If you checked off any problems, how difficult have these problems made it for you to do your work, take care of things at home, or get along with other people?	1.37 ± 0.56	1.64 ± 0.88	0.053

^1^ Statistical significance determined by Fisher’s exact tests (depression scoring), or independent samples *t*-tests (scoring per question).
